# Knowledge of HIV and factors associated with attitudes towards HIV among final-year medical students at Hanoi medical university in Vietnam

**DOI:** 10.1186/1471-2458-14-265

**Published:** 2014-03-20

**Authors:** Michael Platten, Ha N Pham, Huy V Nguyen

**Affiliations:** 1 Department of Public Health Sciences, Karolinska Institutet, Stockholm, Sweden; 2 World Health Organization, Hanoi, Vietnam; 3 Department of Health Management and Organization, Institute for Preventive Medicine and Public Health, Hanoi Medical University, Hanoi, Vietnam

**Keywords:** HIV, Vietnam, Medical students, Knowledge, Attitude

## Abstract

**Background:**

The success of HIV care strongly depends upon skills of the healthcare worker. Vietnam has a punitive history towards HIV and even though this has changed recently, persons living with HIV are still facing discrimination. The objective of this paper is to assess the gaps in knowledge of HIV and factors associated with discriminatory attitudes towards persons living with HIV among medical students in order to improve medical training.

**Methods:**

In a cross-sectional quantitative study using a structured questionnaire, 200 final-year medical students at Hanoi Medical University were approached for data collection in May of 2012. Descriptive statistics (percentages) were used to present four HIV knowledge tests. Linear regression models were examined to highlight factors that are associated with general attitudes towards HIV and attitudes towards HIV in a clinical setting.

**Results:**

Although students performed overall well in the knowledge category of HIV discrimination and stigma, there were several gaps in knowledge of HIV, including the categories of HIV-related basic sciences, prevention, and care and treatment. Knowledge of stigma and discrimination was a significant positive predictor of *General non-prejudicial attitude to HIV and AIDS* (β = 0.186, P < 0.01) and *Non-discriminatory attitude to HIV and AIDS at work* (β = 0.188, P < 0.01). Training on methadone treatment was found to be a significant positive predictor (β = 0.168, P < 0.05) while family size was negatively associated (β = -0.170, P < 0.05) with *General non-prejudicial attitude to HIV and AIDS*.

**Conclusions:**

The study suggests a need for incorporating HIV training into the core curricula for medical students. As persons who inject drugs carry a proportionately high burden of HIV in Vietnam, it is also important to include methadone training for students.

## Background

It has been demonstrated multiple times that the success of a health system depends critically on the size, skills and commitment of the health workforce. Unfortunately, today’s health systems are in need of improvement, evidenced by a lack of trained health professionals [1]. This disparity becomes even more evident in the case of HIV. The World Health Organization (WHO) has confirmed the importance of the HIV health workforce in order to achieve the development goals and has stressed the need to increase the number of HIV health workers and expand their capacity to deliver HIV services [2].

Vietnam has a population of about 87 million [3]. By the end of 2011, HIV cases had been found in all 63 provinces, 98% of the districts, and 77% of the communes [4]. The overall adult HIV prevalence (age 15–49) is 0.47%, estimated to about 280,000 persons in 2012 [5]. Vietnam belongs to the group of countries with a high ratio of health workers per population (defined as > 5 health workers per 10 000 population). The number of health workers per 10,000 increased from 29.2 in 2001 to 34.4 in 2008 [6]. Despite this there are limitations in quantity and quality of health workers which inhibit the expansion of quality practices in provincial and district levels. In order to alleviate some of the tensions caused by lack of proper training, the Ministry of Health (MoH)’s Joint Annual Health Review has recommended that improvements in health worker quality should be achieved through investments in training facilities, improving quality of instructors, reforming curricula, materials, teaching methods, and strengthening practice facilities [6]. However, it is critical to determine where gaps in knowledge exist in order to materialize this recommendation in a useful fashion. This research, which is the aim of this paper, has not yet to our knowledge been published for a medical school in Vietnam.

Since the 1990s and early 2000s, Vietnam has moved from punitive HIV policies towards a more rights-based approach of HIV prevention and care. This included legalizing harm reduction programs like needle and syringe exchange programs [7]. However, even now over 20 years since the first HIV case in Vietnam has been reported, persons living with HIV (PLHIV) are still facing discrimination. The Vietnam AIDS progress report from 2010–2011 still documented cases of children being denied entry to school, PLHIV being removed from their positions, discrimination towards men who have sex with men (MSM), and injecting drug users (IDUs) and sex workers (SWs) in places without proper treatment services [4]. The Joint Annual Health Review in Vietnam acknowledges that there are many problems and limitations in care and prevention treatment of PLHIV between medical facilities [8]. Due to these circumstances, it is highly important to evaluate the training of medical students, the future’s physicians, in Vietnam in order to pinpoint gaps in knowledge and factors associated with discriminatory attitudes towards PLHIV.

## Methods

### Setting

The study was carried out as part of a larger project supported by Family Health International attempting to identify HIV/AIDS training needs for preventive medicine doctors and general medical doctors in Vietnam. The study was conducted at Hanoi Medical University which is located in Hanoi, in Northern Vietnam. This is one of the leading medical universities among eight medical universities in Vietnam and it is composed of 72 departments with more than 1000 lecturers, researchers, and staff. There are almost 2500 under-graduate and 2500 post-graduate students trained each year, majority of which are trained in clinical and sub-clinical fields, as well as preventive medicine and public health. As a leading medical university it is well situated to undertake this proposed assessment. The study was performed in May of 2012.

### Participants

Final-year medical students were chosen because they had almost completed the medical program and would thus provide a good indication of the knowledge of HIV and attitude towards HIV among newly graduated medical doctors. 200 out of the 500 final-year medical students at HMU were randomly selected using a computer-based random generator. The researchers then went to the class and asked the professor for permission to meet with the selected students. The students were informed of the study and all of the selected students agreed to participate in the study. There are two main medical tracks at HMU; preventive medicine and general medicine. General medicine students account for the largest portion of medical students. At the conclusion of the survey each participant was thanked for their contribution and received 50,000 VD, approximately 2.5 USD, to compensate their time.

### Ethics

The study protocol was approved by both the Institutional Review Board of the Centre for Disease Control in Atlanta and by the Hanoi Medical University Institutional Review Board. Participants were informed of the research objectives and purposes prior to the survey. Participants were advised that they could withdraw from the study at any point without penalty. They were also advised that the data would be handled confidentially and results would be reported at an aggregated group rather than at an individual level.

### Instrument

The paper questionnaires were administered and collected onsite by a researcher of the assessment team. The questionnaires were given in Vietnamese and then translated into English by a medical doctor with over 20 years of translating experience.

The questionnaire had three main components: demographic data information, HIV knowledge tests, and scales of attitude towards HIV. Demographic data were collected with a series of closed questions with forced responses. Most items in the knowledge tests of the questionnaire included an option for “other” type responses.

Many of the items used in the questionnaire were adopted from previous studies of Family Health International 360 (FHI360), Li et. al [9], and other studies done in Vietnam. Four categories of knowledge tests were analyzed from the questionnaire: 1) HIV basic sciences (epidemiology-virology-immunology); 2) HIV prevention knowledge; 3) HIV care and treatment knowledge and 4) HIV discrimination and stigma knowledge.

Each knowledge test contained between five and eight questions which were analyzed. There were three different types of clearly labeled questions in the questionnaire.

I) Enumeration: this type of question was not included in the analysis due to its unstructured nature and poor reflection of knowledge.

II) Multiple choice question with multiple answers: this type of question was followed by four to nine choices, of which the student then had to choose ‘yes’ or ‘no’ for each option. These questions were included in the analysis.

III) Multiple choice question with only one answer: This type of question was followed by four to nine choices, but of which the student only had to choose one correct answer. These questions were also included in the analysis.

The two attitudinal scales were adopted from a previous study by Li et. al [9], which they had adopted from the HIV and AIDS-related Stigma and Discrimination Indicators Development Workshop held by USAID [10]. The two attitudinal scales were used to model and find factors associated with stigma and discrimination: *General non-prejudicial attitude to HIV and AIDS* and *Non-discriminatory attitude to HIV and AIDS at work*. For the attitudinal questions a likert scale was used, with each likert item using a 5- point scale: 1 (Strongly Disagree), 2 (Slightly Disagree), 3 (Neutral), 4 (Slightly Agree), and 5 (Strongly Agree).

The original questionnaire contained six umbrella categories encompassing 49 domains with four to nine items each. Two of these categories were not analyzed in this article: knowledge of drug use, and palliative care and nutrition. These sections were not included due to their wider scope and potential for diluting the more important core subjects that are discussed in this article. There were also a few attitudinal scales omitted due to their low cronbach alpha values which occluded them from further analysis.

### Analysis

Data were entered using ACCESS and then transferred into SPSS version 21.0 (SPSS Inc., Chicago, IL, USA) for analysis. Descriptive statistics were used for the knowledge question. For each question, whether it had multiple answers or just a single answers, a percentage reflecting the number of students that chose the correct answer was presented. This allowed for direct analysis and discussion of the students’ knowledge.

For the attitudinal scale *General prejudicial attitude towards HIV and AIDS*, seven items were selected from the original scale and their scores were summed up in such a way that higher scores reflected a more positive attitude. Acceptable reliability was supported by a Cronbach’s alpha value of 0.765.

For the attitudinal scale *Non-discriminatory attitude towards HIV and AIDS at work*, four items were selected from the original scale and their scores were summed up in such a way that higher scores reflected a more positive attitude. Acceptable reliability was supported by a Cronbach’s alpha value of 0.723.

Several regression models were created using 11 available variables taken from the demographic data of the students as well as one variable made from the knowledge test of *Knowledge of HIV discrimination and stigma*. Then the two best (linear regression) models were identified. For the variable of *Knowledge of HIV discrimination and stigma*, the scores from all 20 items were summed up, so that a higher score reflected a higher level of knowledge of HIV discrimination and stigma. Acceptable reliability was supported by a Cronbach’s alpha value of 0.927.

## Results

### Selected characteristics of the sample

Most were females (56%) and the mean age of the sample was 23.9 ± 0.8. Most were trained in general medicine (87.5%). The students had been trained in the following: basic knowledge on HIV (81%), treatment of AIDS patients (49%), drug addiction treatment with methadone (9.5%), voluntary counseling and testing (VCT) for HIV (44%), prevention of mother-to-child HIV transmission (PMTCT) (55.5%), ARV treatment of HIV-infected patients (36%), nutrition of HIV-infected patients (23.5%), and other (2.5%).

### Knowledge

#### HIV basic sciences (Epidemiology, Virology, Immunology)

The students’ knowledge of HIV basic sciences (epidemiology, virology, and immunology) varied greatly between questions as presented in Table 1. Medical students demonstrated several areas of deficit in knowledge. Overall, a few strengths and several weaknesses in knowledge have been identified within the category of *HIV basic sciences (epidemiology, virology, and immunology)*.

**Table 1:** HIV basic sciences (Epidemiology, Virology, Immunology)

**Variable**	**N (%)**
When does mother-to-child HIV transmission happen	
All three stages	128 (64.0)
Groups where HIV is most prevalent	
Persons who inject drugs	185 (92.5)
Female sex workers	184 (92.0)
Men who have sex with men	122 (61.0)
How long can HIV survive outside body	
A few days	29 (14.5)
HIV can be destroyed by which sterilizing method	
Regular disinfectants	100 (50.0)
UV rays/gamma rays	134 (67.0)
The main target cells that HIV infects and depletes	
TCD4 lymphocytes	82 (41.0)
How does HIV affect T_CD4_ lymphocytes	
Changes in TCD4 lymphocytes count	146 (73.0)
Infection risk of HBV&HCV compared to HIV	
Hepatitis B Higher	92 (46.0)
Hepatitis C Higher	77 (38.5)
Which body fluids transmit HIV	
Blood	200 (100.0)
Semen	176 (88.0)
Vaginal Fluid	175 (87.5)
Breast milk	165 (82.5)

#### HIV prevention

In the category of HIV prevention, many students correctly answered several questions, but still showed some gaps in knowledge as shown in Table 2. Most students have knowledge of how certain modes of HIV transmission can be prevented and 82.0% of students correctly answered that there is no HIV vaccine. Students appeared unclear on the objective of voluntary counseling and testing (VCT). In the category of HIV prevention students performed well in some questions but lacked knowledge in others, especially on the role of VCT.

**Table 2:** HIV prevention knowledge

**Variable (N = 200)**	**N(%)**
HIV can be prevented by	
Condom use during sexual intercourse	189 (94.5)
Do not use shared syringes	198 (99.0)
Safe blood transfusion	199 (99.5)
No HIV vaccine available for prevention	164 (82.0)
Circumstances of occupational exposure	
Blood/body secretions onto scratches/wounds	189 (94.5)
Skin puncture by needles	199 (99.5)
How should occupational HIV exposure be handled	
On-the-spot treatment of the injury	173 (86.5)
Assess HIV exposure risk	156 (78.0)
Determine HIV status of the source person	158 (79.0)
Test for HIV 03–06 months after exposure	192 (96.0)
ARV treatment for the exposed person	166 (83.0)
Have ever heard about HIV harm reduction programs	122 (61.0)
The role of Voluntarily Counseling and Testing	
Provide HIV test results	92 (46.0)
Help PLHIV better understand available services	102 (51.0)
Reduce and mitigate discrimination	75 (37.5)
Reduce risk behaviors for people testing negative for HIV	81 (40.5)
Reduce risk of transmissible behavior of PLHIV	107 (53.5)
Provide psychological support for PLHIV	102 (51.0)
Provide support in the disclosure of HIV status	48 (24.0)
Enhance treatment adherence	81 (40.5)
When is post-exposure ARV treatment is effective	
As soon as possible if the source person is known to have HIV	178 (89.0)

#### HIV care and treatment

In the category of HIV care and treatment, students’ scores varied but were often low as demonstrated in Table 3. The results varied from 28.0% to 88.0% when asked about what can be done to enhance ARV treatment adherence, with only 28.0% identifying methadone therapy for heroin addicts living with HIV. Knowledge of available support services for ARV treatment was particularly low for methadone therapy for heroin users living with HIV (32.0%). The scores were overall low in the category of HIV care and treatment.

**Table 3:** Knowledge of HIV care and treatment

**Variable (N = 200)**	**N(%)**
Most common co- infections/syndromes in Vietnam	
Tuberculosis (TB)	180 (90.0)
Oral mycosis	118 (59.0)
Wasting syndrome/Chronic fatigue syndrome	72 (36.0)
What qualifies a PLHIV for ARV treatment	
Clinical and para-clinical conditions	128 (64.0)
Main cause of ARV drug resistance	
Non adherence to treatment	91 (45.5)
How to enhance ARV treatment adherence	
Regular treatment monitoring and supervision	176 (88.0)
Challenges facing workers and provision of counseling	144 (72.0)
Community outreach of healthcare services	115 (57.5)
Peer group’s support	135 (67.5)
Directly Observed Treatment (DOT)	110 (55.0)
Methadone therapy for Heroin addicts living with HIV	56 (28.0)
Education/counseling on treatment adherence	156 (78.0)
Available support services related to ARV treatment	
Counseling and support for treatment adherence	178 (89.0)
Palliative care	111 (55.5)
Home-based/community-based care	134 (67.0)
Preventive care and opportunistic infection treatment	167 (83.5)
Peer group’s support	134 (67.0)
Methadone therapy for heroin users living with HIV	64 (32.0)

### HIV stigma and discrimination

Knowledge of HIV discrimination and stigma was overall high among the medical students as demonstrated in Table 4. Students’ scores ranged from 75.5% to 96.0% in regards to the signs of discrimination. Most students identified the main causes of HIV discrimination in Vietnam: lack of HIV knowledge (89.0%), fear of HIV (92.5%), and the connection of HIV with “social evils” (81.0%). The students correctly identified how discrimination/stigma affects the community with correct rates of 78.5%- 90.0%. Overall, students scored well in the category of knowledge of HIV discrimination and stigma.

**Table 4:** Knowledge of HIV discrimination and stigma

**Variable (N = 200)**	**N(%)**
Signs of discrimination	
Avoidance: avoid touching, proximity, etc.	192 (96.0)
Denial: denied housing, job loss, etc.	169 (84.5)
Isolation: isolated area in hospitals, etc.	178 (89.0)
Gossip from the community	179 (89.5)
Loss of status within household and community	155 (77.5)
Loss of access to essential resources	151 (75.5)
Main causes of stigma against PLHIV	
Lack of knowledge about HIV	178 (89.0)
Fear of HIV	185 (92.5)
Connection of HIV to “social evils”	162 (81.0)
How does stigma affect PLHIV?	
Self-discrimination	169 (84.5)
Job loss or inability to find employment	176 (88.0)
Difficulties in accessing social support services	177 (88.5)
Hiding HIV status	181 (90.5)
How does stigma affect HIV patients’ family	
Family members lose access to social support services	156 (78.0)
Family income is affected- limited employment	170 (85.0)
Family members also become victims of stigma	178 (89.0)
Relationships within the households are affected	171 (85.5)
How does stigma affect the community/society	
Stigma increases HIV transmission risks	157 (78.5)
Wasting resources due to PLHIV don’t want to access intervention programs	178 (89.0)
Stigma destroys traditional values (sense of belonging)	180 (90.0)

### Factors associated with attitudes towards HIV

Two attitudinal scales, adopted from a study by Li et. al, were used to create models of associated factors. The resulting two models both demonstrated that higher knowledge of HIV stigma and discrimination correlate with more positive attitudes towards HIV.

Three variables were significantly associated with the outcome of *General non-prejudicial attitude to HIV and AIDS* as shown in Table 5. Knowledge of HIV stigma and discrimination was significantly and positively associated (Beta = .186, P < .01). Training on methadone treatment was also significantly and positively associated (Beta = .168, P < .05). The number of family members was significantly and negatively associated (Beta = -.170 P < .05).

**Table 5:** Factors associated with attitude to HIV

**Independent variables**	**Standardized regression coefficients (beta)**	**Index of model fit**
***Factors associated with general non-prejudicial attitude to HIV/AIDS***		
Knowledge of stigma and discrimination (KSD)	.186**	Adjusted R^2^ = .087
		F-test = 6.969***
Training on methadone treatment (TMT)	.168*	
Number of family members	-.170*	
***Factors associated with non-discriminatory attitude to HIV and AIDS at work***		
Knowledge of stigma and discrimination (KSD)	.188**	Adjusted R^2^ = .030
		F-test = 7.247**

Note: *P < 0.05; **P < 0.01; ***P < 0.001.

Only one variable was significantly associated with *non-discriminatory attitude to HIV and AIDS at work*, as shown in Table 5. Knowledge of HIV stigma and discrimination was significantly and positively associated (Beta = .188 P < .05).

## Discussion

The goals of this study were to discover where the potential gaps in HIV knowledge exists and to explore factors associated with HIV-related stigma. This information would help unveil how the medical education could be improved in Vietnam. In this study it was found that students had specific shortfalls in the knowledge categories of *HIV basic sciences*, *HIV prevention* and *HIV care and treatment*. Conversely, they scored relatively high in the knowledge category of *HIV discrimination and stigma*. Through the linear regression models it was identified that *knowledge of HIV stigma and discrimination* and *training on methadone maintenance therapy* were both significant and positive predictors of *general non-prejudicial attitude to HIV and AIDS,* while the *number of family members* was a significant and negative predictor of *general non-prejudicial attitude to HIV and AIDS.* The outcome of the second model showed that only *knowledge of HIV stigma and discrimination* was a significant and positive predictor of *non-discriminatory attitude to HIV and AIDS at work.*

### Knowledge of HIV

Although improvements have been made in increasing knowledge and raising awareness, gaps of HIV knowledge remain prevalent in various populations. Most literature supports that even though there may be specific areas of strength, there are gaps of HIV knowledge in: general populations [11], medical students [12,13], and healthcare workers [9,14-17]. Comparing knowledge levels between different studies has some limitations due to different questionnaire designs, but some comparisons are possible. For example, in an older study by Reza Najem and Okaye Okuzu [12], it was found that both Benin and U.S. medical students had misperceptions about maternal-infant transmission, 26% to 51% believing it was invariable. The students had high levels of knowledge of basic transmission routes like sex and shared needles. Over 90% of both student populations knew that there was no effective vaccine against HIV. Both groups had high knowledge of PWID as a high risk-group [12]. These results were similar to the results of our study, though regarding the question of an HIV vaccine, 18% of HMU students believed one existed. The HMU students also struggled with maternal-infant transmission (64.0% answered correctly) but the students had a high level of knowledge of PWID as a high-risk group (92.5%). Another study comparing fourth-year medical students at the University of Zagreb, Croatia, showed comparable results to our study. Medical students at Zagreb were able to identify transmission routes of HIV, yet only 31.6%-36% favored voluntary testing, and only 46%-61% of students knew that the probability of a newborn acquiring HIV infection from an infected mother was between 15%-40% [13]. Even though the latter two questions were not directly asked to the HMU students in our study, the results exhibit a lack of knowledge which was present in these categories among the HMU students. Another study comparing the knowledge of HIV among last year medical students with pharmacy students in Malaysia showed that even though medical students had slightly higher scores than the pharmacy students there existed gaps of HIV knowledge. For example, the knowledge of HIV transmission was very high, even for maternal-infant transmission (95.2%-99%) but knowledge was rather low regarding antiretroviral post-exposure prophylaxis (48.6%) and drug treatment (47.6%) [14]. HMU students had high knowledge of HIV transmission routes through sex and needle sharing but relatively low knowledge about maternal-infant transmission (64.0%). Most of the HMU students knew that an exposed person should be treated with antiretroviral prophylaxis treatment (83.0%). Yet another study of students of health-related subjects in Yemen concluded that students had a moderate level of HIV knowledge with 67.6% of students answering all the questions correct. Most students knew that HIV could be transmitted by sexual intercourse without a condom (82.3%), from syringes (87.5%), from infected blood (71.8%) and from mother to child (80.7%). However, the students from Yemen struggled with the signs and symptoms of AIDS (37.8%-62.1%) [15]. Again this shows the inconsistency of knowledge about HIV/AIDS among students studying health-related fields. It is difficult to generalize the results among these studies since the studies used different questionnaires; however, it is evident that a knowledge deficit exists among medical and health-related students in various global settings. The deficiency of knowledge as demonstrated in our study among HMU medical students suggests the need for improvement in HIV training.

### Factors associated with HIV-related stigma

In our study we found that knowledge of HIV-related stigma and training on methadone treatment were predictors of less general prejudicial attitudes towards HIV, while a larger number of family members predicted more general prejudicial attitudes towards HIV. Knowledge of HIV stigma and discrimination was also a predictor of less discriminatory attitudes towards HIV at work.

The literature pinpoints an array of factors that are associated with HIV-related stigma. The most prevalent factor was a lack of knowledge in regards to HIV [13,18-22]. However, several other factors are also identified: lack of exposure to PLHIV [13,21,23-25], stigma towards high-risk groups like MSM, PWID, and SW [19,20,25], lack of HIV training [9,20,26], fear of contagion [9,20,27], age [21], and gender [20]. It is clear from the literature and supported by our study that greater knowledge of HIV leads to more positive attitudes towards HIV-related stigma. This is why it is important to focus on improving the educational platform in order to provide sufficient HIV education for students while in medical school. Our results and the literature also support that HIV training helps reduce HIV-related stigma. It makes sense that in Vietnam, where the majority of PLHIV are PWID [28], that methadone treatment training will reduce prejudicial attitudes towards HIV-related stigma. Knowing that poor knowledge is a predictor of stigmatizing views towards PLHIV, it is important to discuss what can be done to improve the knowledge and training for medical school students at HMU.

The third factor regarding increased family size predicting more prejudicial attitudes towards HIV was an unexpected finding that needs further research as there is scarce literature that directly supports or refutes this. However, some assumptions may be extracted from this result as well as based on the social context of developing countries, including Vietnam. Often more family members are associated with a lower socioeconomic status as well as a lower level of education. This in turn could lead to a lower level of HIV knowledge which we have found leads to a higher level of HIV-related stigma. Thus the stigmatizing pressure may be greater from students that come from larger families. This is an interesting factor that may be important to take into account when designing a curriculum for the HMU medical students, by highlighting the influence and role of family in possible discriminatory attitudes.

### How to reduce HIV stigma and discrimination?

There are many ways to reduce HIV-related stigma and discrimination. As physicians are part of the general society, their attitudes and perceptions are influenced by societal norms. If HIV were viewed as a disease without stigma, healthcare providers would be more at ease with treating PLHIV. Thus stigma reduction in the population will lead to a stigma reduction among health care providers [29]. Since poor knowledge of HIV is a predictor of stigmatizing views towards PLHIV, there is a strong need to develop effective educational intervention that increase HIV knowledge [30]. This can include education and communication in mass media to improve the public image of HIV and availability of services [31].

It has been noted that it is important to address HIV stigma at both an individual and institutional level [9]. The Vietnam national strategy on HIV/AIDS prevention and control 2010 and 2020 has identified that it is important to cooperate with medical schools in order to improve training programs by integrating issues of HIV, stigma and discrimination into the curriculum [11]. Medical school curricula need to be updated to provide future physicians with the skills necessary to fight HIV [16].

A thematic literature review of nursing students’ attitudes towards PLHIV found a study which showed that a specialized HIV training course led to increased knowledge of HIV which in turn resulted in more positive attitudes towards PLHIV [20]. A separate review of Chinese health care providers’ attitudes of HIV showed that a successful training intervention resulted in increased sympathy and willingness to care for PLHIV. Another study showed that as little as a one-time lecture increased self-reported willingness to work with PLHIV [18]. A study of physicians in Barbados concluded that it is essential for physicians to receive educational programs that highlight how their prejudicial views affect PLHIV’s health seeking behavior. It was also determined that physicians should receive training on the skills needed to work with PLHIV [16]. Treat, Train, Retain: The AIDS and health workforce plan written by the WHO [2], recommended that healthcare workers receive both pre-service and in-service training. Pre-service training means integrating HIV services into the curriculum as well as increasing the number of graduates and facilities [2]. A literature review of interventions to reduce HIV stigma found that contact with PLHIV for trainees may be the most promising approach, though it must also be supplemented with improved understanding of the disease itself [23]. The literature as well as our study emphasizes the need for better HIV knowledge among healthcare workers. In conclusion, it is important to have an integrated and multidirectional approach which can provide relevant knowledge, while removing fear and negative views towards HIV [21]. This can be achieved by making HIV a recurring topic throughout the curriculum and facilitating personal contact with PLHIV in addition to pre-service and in-service training.

### Limitations

This data would be representative of last-year medical students at Hanoi Medical University. Even though HMU is a leading medical university in Vietnam, the results may not be generalizable to medical school students of the whole country. Additionally, the adjusted coefficient of determination is low for the models (8.7% and 3.0%) suggesting that there may be other important factors that could influence the prejudicial attitudes towards HIV among the medical students. As this is a cross-sectional study, it may preclude the order of causality between factors and attitudes towards HIV. Therefore it is important that future research should consider longitudinal design to confirm the temporal relation.

## Conclusions

Even though there are several strengths, the overall knowledge of HIV among medical students in the categories of *HIV basic science*, *HIV prevention* and *HIV care and treatment* have several shortcomings. This needs to be improved by properly integrating HIV training into the medical school curriculum. HIV training needs to be prioritized and recurring with special emphasis placed on the aforementioned categories. Improved knowledge among physicians will lead to more positive attitudes and less HIV-related stigma.

It has been shown in our study, as well as others, that improving knowledge of HIV will have a positive effect on the attitudes towards PLHIV. Alleviating the stigma around the high-risk groups will also lead to more positive attitudes toward HIV.

In this study we also showed that training in methadone treatment was a significant and positive predictor of decreased general prejudicial attitude towards HIV. This is particularly important in Vietnam where PWID account for the largest portion of PLHIV. An integrated approach throughout the curriculum with facilitated personal contact with PLHIV in addition to pre-service and in-service training will help ensure that the knowledge endures.

## Competing interest

The authors have declared no conflict of interests.

## Authors’ contributions

NVH and PNH conceptualized the study. MP, NVH, and PNH all contributed to data analysis and writing the manuscript. All authors read and approved the final manuscript.

## Authors’ information

PNH is a PhD at Karolinska Institutet and is employed at WHO Vietnam. MP has a Master of Global Health at Karolinska Institutet. NVH has a PhD in public health in Australia and is a lecturer at Hanoi Medical University Unit in Hanoi, Vietnam.

## Pre-publication history

The pre-publication history for this paper can be accessed here:

http://www.biomedcentral.com/1471-2458/14/265/prepub
