# Correction: knowledge of HIV and factors associated with attitudes towards HIV among final-year medical students at Hanoi medical university in Vietnam

**DOI:** 10.1186/1471-2458-14-576

**Published:** 2014-06-09

**Authors:** Michael Platten, Ha N Pham, Huy V Nguyen, Nhu T Nguyen, Giang M Le

**Affiliations:** 1 Department of Public Health Sciences, Karolinska Institutet, Stockholm, Sweden; 2 World Health Organization, Hanoi, Vietnam; 3 Department of Health Management and Organization, Institute for Preventive Medicine and Public Health, Hanoi Medical University, Hanoi, Vietnam; 4 Family Health International 360, Hanoi, Vietnam; 5 Department of Epidemiology, Institute for Preventive Medicine and Public Health, Hanoi Medical University, Hanoi, Vietnam

## Correction

After the publication of this work [1], we became aware that two authors had not appeared in the authorship list. This is because during the article write-up and review process, we forgot to include some other authors who at the beginning stage of the research also contributed greatly to the conceptualization and design of the research. This caused the incomplete authorship in the article.

**The updated author list is:** Michael Platten, Ha N Pham, Huy V Nguyen, Nhu T Nguyen & Giang M Le.

**The updated authors’ contributions are:** NTN, LMG and NVH conceptualized and designed the study. MP wrote the manuscript. NVH and PNH both contributed to data analysis and writing the manuscript. NTN and LMG applied for ethical approval for this study. All authors read, reviewed and approved the final manuscript.

## Pre-publication history

The pre-publication history for this paper can be accessed here:

http://www.biomedcentral.com/1471-2458/14/576/prepub
